# Refining breast cancer biomarker discovery and drug targeting through an advanced data-driven approach

**DOI:** 10.1186/s12859-024-05657-1

**Published:** 2024-01-22

**Authors:** Morteza Rakhshaninejad, Mohammad Fathian, Reza Shirkoohi, Farnaz Barzinpour, Amir H. Gandomi

**Affiliations:** 1 Industrial Engineering Department, Iran University of Science and Technology, Hengam Street, Tehran, 1684613114 Tehran Iran; 2 Cancer Biology Research Center, Cancer Institute, Imam Khomeini Hospital Complex, Tehran University of Medical Sciences, Keshavarz Boulevard, Tehran, 1419733141 Tehran Iran; 3 Faculty of Engineering and Information Technology, University of Technology Sydney, Ultimo, 2007 NSW Australia; 4 University Research and Innovation Center (EKIK), Óbuda University, Budapest, 1034 Hungary

**Keywords:** Breast cancer, Machine learning, Biomarker discovery, Ensemble method, Hybrid metaheuristic algorithm

## Abstract

Breast cancer remains a major public health challenge worldwide. The identification of accurate biomarkers is critical for the early detection and effective treatment of breast cancer. This study utilizes an integrative machine learning approach to analyze breast cancer gene expression data for superior biomarker and drug target discovery. Gene expression datasets, obtained from the GEO database, were merged post-preprocessing. From the merged dataset, differential expression analysis between breast cancer and normal samples revealed 164 differentially expressed genes. Meanwhile, a separate gene expression dataset revealed 350 differentially expressed genes. Additionally, the BGWO_SA_Ens algorithm, integrating binary grey wolf optimization and simulated annealing with an ensemble classifier, was employed on gene expression datasets to identify predictive genes including TOP2A, AKR1C3, EZH2, MMP1, EDNRB, S100B, and SPP1. From over 10,000 genes, BGWO_SA_Ens identified 1404 in the merged dataset (F1 score: 0.981, PR-AUC: 0.998, ROC-AUC: 0.995) and 1710 in the GSE45827 dataset (F1 score: 0.965, PR-AUC: 0.986, ROC-AUC: 0.972). The intersection of DEGs and BGWO_SA_Ens selected genes revealed 35 superior genes that were consistently significant across methods. Enrichment analyses uncovered the involvement of these superior genes in key pathways such as AMPK, Adipocytokine, and PPAR signaling. Protein-protein interaction network analysis highlighted subnetworks and central nodes. Finally, a drug-gene interaction investigation revealed connections between superior genes and anticancer drugs. Collectively, the machine learning workflow identified a robust gene signature for breast cancer, illuminated their biological roles, interactions and therapeutic associations, and underscored the potential of computational approaches in biomarker discovery and precision oncology.

## Introduction

Breast cancer remains one of the foremost health challenges globally, consistently accounting for a significant fraction of cancer diagnoses and mortalities among women. The World Health Organization (WHO) statistics underscore the escalating prevalence of breast cancer (BC) cases and the associated mortalities [1]. The pivotal role of early detection cannot be overstated: it not only dramatically improves the prognosis but also potentially reduces mortality rates [2]. In this light, crafting precise and accurate predictive models for BC detection is of paramount importance, bridging the gap between early diagnosis and effective therapeutic interventions.

Despite technological advancements, the field of BC research grapples with numerous challenges, primarily in the identification of accurate biomarkers [3]. The high rate of false positives in BC detection is a considerable impediment, necessitating more reliable predictive models [4]. Another significant barrier is the scarcity of comprehensive datasets. To ameliorate this issue, integrating existing datasets can be invaluable [5]. Machine learning (ML) offers a transformative solution to these challenges by facilitating the use of combined gene expression datasets to enhance the accuracy and reliability of BC prediction.

Embarking on an interdisciplinary approach, this study melds ML and optimization algorithms with systems biology to identify genes intrinsically linked to BC. Through an exhaustive meta-analysis of the Gene Expression Omnibus (GEO) datasets, we harnessed techniques to tease out genes that starkly demarcate BC from normal samples. The curated gene signatures were then juxtaposed with differentially expressed genes and subjected to rigorous Gene Ontology (GO) and Kyoto Encyclopedia of Genes and Genomes (KEGG) enrichment analyses. This culminated in the formulation of a state-of-the-art feature selection (FS) algorithm, enhancing the efficiency and accuracy of biomarker identification. This interdisciplinary effort extends beyond mere prediction, delving into the identification of superior genes that offer promising insights into the mechanisms of the disease and potential therapeutic targets.

The primary aim of this paper is to develop and validate a novel predictive model for BC detection that surpasses current methods’ limitations. This research, to the best of our knowledge, is the first to apply a unique combination of hybrid metaheuristic algorithms and ensemble models to gene expression datasets, targeting the discovery of biomarkers with unparalleled precision. The successful identification of key genes in this study marks a significant advancement in comprehending BC, opening up new possibilities for drug-gene interaction analysis and the development of personalized medicine strategies.

The organization of this paper is meticulously structured to guide the reader through the intricate processes and findings of the study. Following this introduction, in section “Literature review” conducts a critical review of the pertinent literature. In section “Materials and methods” delineates the materials and methods, encapsulating data procurement, FS protocols, ensemble method formulation, and drug-gene interaction analysis. In section “Results” unveils the empirical results, including differential gene expression analysis and the identification of superior genes. In section “Discussion” interprets these findings within the broader context of biomarker discovery and precision oncology. The paper reaches its culmination in section “Conclusion”, where the implications of the study for the future of BC detection and treatment are synthesized.

## Literature review

Breast cancer is a multifaceted disease with a genomic footprint that can be deciphered for early detection and treatment strategies. In the quest for precision medicine, ML algorithms have become indispensable tools in analyzing complex biomedical data, such as gene expression profiles, to identify potential biomarkers for diseases such as BC [6].

Unlike imaging or clinical data, gene expression data offer a dynamic picture of the cellular mechanisms at play during oncogenesis [7]. High-throughput technologies such as microarrays and RNA sequencing facilitate a holistic view of gene activity across the genome [8]. The GEO and ArrayExpress databases exemplify resources that have democratized access to such data, enabling extensive research into gene function and regulation [9].

The discovery of gene expression biomarkers is paramount for diagnostic and prognostic advancements in BC [10]. Differential gene expression analysis serves as a cornerstone methodology, distinguishing between normal and diseased states by identifying genes with significantly altered expression [11].

As the volume and complexity of gene expression data burgeon, ML offers a suite of algorithms capable of deciphering intricate patterns within the data [12]. Studies have utilized various ML approaches, such as decision trees (DT) [13], neural networks (NN) [14], support vector machines (SVM) [15], logistic regression (LR) [16], and random forests (RF) [17], alongside more recent innovations in deep learning (DL) [18, 19] and ensemble learning [20, 21] methods such as extreme gradient boosting (XGBoost) [5] and adaptive boosting (AdaBoost) [22], to identify significant biomarkers in BC.

The preprocessing of gene expression data, including normalization [23] and FS [24], is vital to ensure the reliability of subsequent analyses. The Robust Multiarray Average (RMA) is often employed for its robustness in normalization and expression calculation [25]. Feature selection is a critical step to highlight relevant genes and can be addressed through various methods, including filter, wrapper, embedded, and dimensionality reduction techniques [26].

Merging and combining gene expression datasets enhance the power and scope of analysis, enabling a comprehensive view across varied conditions, treatments, and populations, thus improving the generalizability and robustness of the findings [27]. Such integration is essential to address batch effects through normalization, which is crucial when datasets from different sources are combined [5]. Moreover, a larger dataset provides a more substantial basis for FS methods to uncover informative genes, leading to more accurate models of gene expression [28]. This process not only corroborates the findings across different studies, strengthening the reproducibility of research but also significantly advances the field of personalized medicine and our understanding of complex diseases.

The combinatorial challenge of FS has been approached with metaheuristic algorithms, which offer powerful solutions to NP-Hard problems through intelligent search strategies [29]. Algorithms such as genetic algorithms (GA) [30], simulated annealing (SA) [31], whale optimization algorithm (WOA) [32], grey wolf optimization (GWO) [33], and particle swarm optimization (PSO) [20] have been utilized across various high-dimensional datasets, demonstrating their efficacy in identifying relevant features.

Recent advancements in FS have seen a growing interest in hybrid methods that combine the strengths of different metaheuristic algorithms. These hybrid approaches aim to overcome the limitations of individual algorithms by integrating their unique search mechanisms. For instance, the fusion of GA with artificial bee colony (ABC) has shown promising results in navigating complex search spaces more effectively [34]. Another notable hybrid method involves combining the spotted hyena optimizer (MOSHO) with the salp swarm algorithm (SSA), which enhances the ability of FS in high-dimensional datasets [35]. A novel framework, introducing CS-GA and ABC-CS, combines the cuckoo search (CS) algorithm with ABC and GA to enhance both exploitation and exploration, thus achieving a balanced search process [36]. These hybrid methods are not only capable of handling the high dimensionality of datasets but also exhibit improved performance in terms of accuracy and computational efficiency in FS tasks.

In the realm of FS, the convergence of hybrid optimization techniques with advanced ML methodologies is forging innovative pathways. Notably, in these hybrid models, the use of ensemble learning frameworks [20] as objective functions for metaheuristic algorithms is an emerging and novel approach. This method adeptly refines the search strategy of metaheuristic algorithms to focus on enhancing the predictive accuracy and generalization of ensemble models [37], which are particularly effective in addressing the challenges of imbalanced and high-dimensional data. The result is a more refined FS that is inherently aligned with the core strengths and requirements of the ensemble methods [38].

Following the identification of biomarkers, their functional interpretation is enabled through enrichment analyses, utilizing resources such as GO [39] and KEGG [40]. These tools provide insights into gene functions and pathways, allowing for a deeper understanding of the biological significance behind the differential gene expression observed.

Upon analyzing biomarkers, the investigation of protein-protein interactions sheds light on cellular pathways integral to disease pathophysiology, guiding the creation of targeted therapies [41]. Moreover, comprehending drug-gene interactions becomes essential in formulating personalized medicine approaches, especially in the context of BC treatment [42].

While gene expression data offer tremendous potential in advancing BC research, several challenges persist that can affect the integrity and applicability of the results. Inconsistencies in data preprocessing, such as normalization and FS, can lead to variable and sometimes unreliable outcomes. These issues underscore the need for rigorous and standardized methodologies in handling gene expression data [43]. Additionally, ML models, pivotal in analyzing these complex data, face their own set of challenges. Overfitting remains a primary concern, where models may perform excellently on training data but fail to generalize to new data [5]. This is further compounded by the often intricate nature of these models, leading to issues with interpretability and understanding of the underlying biological mechanisms. Furthermore, the effectiveness of these models is frequently contingent on the availability of large, high-quality training datasets, which are not always accessible or feasible to obtain [44]. These challenges highlight the need for ongoing refinement of ML techniques and data processing methods to enhance the reliability and applicability of gene expression analysis in BC research.

In addressing the complexities and challenges of gene expression data analysis in BC research, our study adopts a holistic and innovative approach. We merged two gene expression datasets to create a robust and diverse data foundation, which is essential for overcoming the limitations of data quality and quantity. This merged dataset is then rigorously processed using standardized methodologies for normalization and FS, ensuring reliable and consistent analysis. Recognizing that reliance on a single, merged dataset might not be fully representative, we have also prepared an additional gene expression dataset, named GSE45827, to test and validate the developed method. To specifically counter issues such as overfitting and to improve model interpretability, our research introduces a novel ensemble learning model, incorporating a specialized weighted voting method. This model is adeptly designed to evaluate the outputs from a hybrid metaheuristic algorithm that synergizes Binary Grey Wolf Optimization (BGWO) and SA. By combining these advanced techniques, our study not only enhances predictive accuracy and FS efficacy but also fills a critical gap in the realm of BC biomarker discovery, paving the way for more precise diagnostic and therapeutic advancements.

## Materials and methods

### Download datasets and preprocessing

The datasets pivotal to this study, specifically GSE10810 and GSE42568, were combined to form a merged dataset. Additionally, the separate dataset GSE45827 was sourced from the NCBI GEO database, all of which are available at www.ncbi.nlm.nih.gov/geo. Corresponding clinical data for these distinct GEO datasets were procured in tandem with the gene expression profiles. The merged dataset comprises a collective total of 179 BC samples and 44 samples earmarked as normal, combining the GSE10810 dataset, which spans 58 samples (31 BC and 27 normal), with the GSE42568 dataset, encompassing 121 samples (104 BC and 17 normal). Separately, the GSE45827 dataset includes 155 samples, segregated into 144 BC samples and a subset of 11 normal samples.

Data preprocessing is pivotal in the realm of microarray data mining. Proper preprocessing not only refines the raw data but also ensures that the resulting data serve as a valid and optimal input for subsequent modeling. Raw datasets were sourced from the GEO repository using the ‘GEOquery‘ package. Subsequent preprocessing, including background correction, normalization, and gene expression calculation, was undertaken using the RMA algorithm.

Furthermore, given that not all probes manifest across all samples, those unexpressed across the entirety of samples were excised. Initial identification was conducted using probe IDs, and gene symbols were subsequently appended based on these IDs. It was noted that certain probe IDs could not be mapped to any gene symbol, warning of their removal. Conversely, instances were observed where multiple probe IDs corresponded to a singular gene symbol. In such situations, to maintain a unified gene expression value, the mean of the expression values of probe IDs sharing the same gene symbol was computed and deemed the final expression value.

All datasets exceed the sample size of 50, ensuring substantial data for rigorous analysis. A consistent platform, GPL570 (Affymetrix Human Genome U133 Plus 2.0 Array), was selected for all three datasets to minimize discrepancies from different platforms and maintain uniformity in gene set examination. Data ingestion was performed using the ReadAffy function from the affy package, followed by normalization using the RMA technique.

### Merge datasets

Merging gene expression datasets, especially when they originate from different sources, presents challenges due to the introduction of nonbiological variations, often referred to as batch effects. In this study, the integration of the two datasets was accomplished using the cbind function in R. However, merging inherently brings forth the aforementioned batch effects.

Adjustments were carried out using the empirical Bayes algorithm to address and rectify these batch effects. This algorithm has been proficiently implemented in the ComBat function, which is part of the SVA package in R. The efficacy of the batch-effect correction achieved by the ComBat transformation was ascertained through Principal Component Analysis (PCA). Specifically, the prcomp function in R was employed to conduct the PCA, which endeavors to encapsulate high-dimensional data into its first two principal components. This dimensional reduction facilitates visualization, for which the ggbiplot package in R was utilized.

After the rigorous phases of preprocessing, batch-effect elimination, and validation, the culmination is a unified dataset. This merged dataset is composed of 179 samples and a comprehensive 10,629 features. Throughout the remainder of this research endeavor, this dataset will be referenced as the “merged dataset”.

### Differential gene expression analysis

Differential Gene Expression (DGE) analysis was pivotal in identifying the differentially expressed genes (DEGs) within the datasets. The analysis was carried out using the renowned limma package in R. The core principle behind the DGE analysis is to pinpoint genes that manifest considerable alterations in their expression levels between contrasting groups, such as diseased versus healthy.

For the purpose of this research, the determination of DEGs was guided by rigorous statistical criteria. A gene was earmarked as differentially expressed if its log-fold change (*logFC*) exceeded 2, indicating significant upregulation or downregulation of its expression. Furthermore, the significance of this differential expression was validated with a stringent *p*-value threshold set at less than 0.05. Only genes satisfying both of these criteria were classified as DEGs in both the merged and GSE45827 datasets.

### Feature selection

As datasets grow in size and complexity, ensuring the relevance of features becomes crucial for the efficient application of ML algorithms [45]. Unnecessary features can not only slow down algorithms but can also compromise their accuracy [46]. Proper FS can significantly enhance algorithm speed and performance [24]. In this study, we focused on identifying a subset of genes from the merged and GSE45827 datasets that are most pertinent to BC prediction. The efficacy of our results hinges on the judicious selection of these genes.

To achieve this, we utilized a fusion of the GWO [47] and SA techniques, employing the BGWO_SA_Ens method. This method stands out due to its incorporation of an ensemble method in its objective function. Essentially, within the BGWO_SA_Ens framework, our goal is to determine a set of genes that maximize the classification accuracy of samples. Further insights into this method can be found in section “BGWO_SA_Ens feature selection algorithm” We also experimented with BGWO_Ens, GA_Ens, LASSO, MCFS_IFS, and mRMR_IFS [48] feature selection methods to compare their results with BGWO_SA_Ens, as detailed in section “BGWO_Ens, GA_Ens, and LASSO feature selection algorithms”.

### BGWO_SA_Ens feature selection algorithm

#### BGWO

The Grey Wolf Algorithm takes inspiration from how wolves hunt. There are four main types of grey wolves: alpha ($\alpha$), beta ($\beta$), delta ($\delta$), and omega ($\omega$) wolves. Grey wolves live in packs and adhere to strict social rules that rank them in the wolf hierarchy. Alpha wolves lead the pack and make decisions. Betas provide assistance to the alphas in making decisions. While deltas obey alphas and betas, they dominate omega wolves. In an effort to reach their prey, wolves attempt to update their position by following the top three wolves. The wolf tries to reach the prey position in the most efficient way possible using this algorithm.

In this algorithm, the location of prey represents the optimal solution, while the location of each wolf represents a candidate solution. In terms of grey wolf solutions, $\alpha$ is the best, $\beta$ is the second best, $\delta$ is the third best, and $\omega$ represents the rest. After the wolves are positioned randomly, their fitness values are calculated. Then, all wolves must update their position relative to those of the top three wolves. Next, the algorithm repeatedly updates the positions of the wolves. This is done by considering the top three performing wolves at each step. During this updating process, the best-performing wolf’s position and its performance score, termed the fitness value, are recorded in every cycle. The main goal of this algorithm is, by the end, to pinpoint the best position that is closest to the prey.

At iteration *t*, $\overrightarrow{X_\alpha }$, $\overrightarrow{X_\beta }$, and $\overrightarrow{X_\delta }$ are the three best solutions. Equation (1) defines $\overrightarrow{A_1}$, $\overrightarrow{A_2}$, and $\overrightarrow{A_3}$ as coefficient vectors.

1 $$\begin{aligned} \overrightarrow{A}=2a\times \overrightarrow{r_1}-a \end{aligned}$$

In Eq. (1), vector $\overrightarrow{r_1}$ is generated by random numbers between 0 and 1. The distance control factor *a* is used to balance the tradeoff between exploration and exploitation, beginning at a high value equal to 2 and decreasing linearly until it reaches 0. The factor *a* is calculated using Eq. (2), where *t* represents the current iteration and $t_{max}$ represents the total number of optimization iterations.

2 $$\begin{aligned} a=2-t\times \frac{2}{t_{max}} \end{aligned}$$

Equations (3), (4), and (5) determine $\overrightarrow{D_\alpha }$, $\overrightarrow{D_\beta }$, and $\overrightarrow{D_\delta }$ as the distance vectors between $\alpha$, $\beta$, $\delta$ and *i*.

3 $$\begin{aligned} \overrightarrow{D_\alpha }= & {} \left| \overrightarrow{C_1}\times \overrightarrow{X_\alpha }-\overrightarrow{X_i} \right| \end{aligned}$$

4 $$\begin{aligned} \overrightarrow{D_\beta }= & {} \left| \overrightarrow{C_2}\times \overrightarrow{X_\beta }-\overrightarrow{X_i} \right| \end{aligned}$$

5 $$\begin{aligned} \overrightarrow{D_\delta }= & {} \left| \overrightarrow{C_3}\times \overrightarrow{X_\delta }-\overrightarrow{X_i} \right| \end{aligned}$$

Equation (6) defines $\overrightarrow{C_1}$, $\overrightarrow{C_2}$, and $\overrightarrow{C_3}$ as coefficient vectors, where vector $\overrightarrow{r_2}$ is generated by random numbers between 0 and 1.

6 $$\begin{aligned} \overrightarrow{C}=2\times \overrightarrow{r_2} \end{aligned}$$

The GWO locates wolves’ positions in continuous space, whereas the BGWO locates them in a hypercube search space with 0 or 1. We have to set some equations to update the positions of the wolves to move them closer or further away from the hypercube.

We can compute $s_{1}^{d}$, $s_{2}^{d}$, and $s_{3}^{d}$ as the continuous step size values using Sigmoid function by Eqs. (7), (8), and (9). Here, *d* is the dimension of the search space.

7 $$\begin{aligned} s_{1}^{d}= & {} \frac{1}{1+\exp (-10(A^{d}\times D_{\alpha }^{d}-0.5))} \end{aligned}$$

8 $$\begin{aligned} s_{2}^{d}= & {} \frac{1}{1+\exp (-10(A^{d}\times D_{\beta }^{d}-0.5))} \end{aligned}$$

9 $$\begin{aligned} s_{3}^{d}= & {} \frac{1}{1+\exp (-10(A^{d}\times D_{\delta }^{d}-0.5))} \end{aligned}$$

A binary step is represented by Eqs. (10), (11), and (12), where *r*3 is a random number between 0 and 1.

10 $$\begin{aligned} bstep_{1}^{d}= & {} {\left\{ \begin{array}{ll} 1 &{} \text { if } s_{1}^{d}\ge r3 \\ 0 &{} \text { else } \end{array}\right. } \end{aligned}$$

11 $$\begin{aligned} bstep_{2}^{d}= & {} {\left\{ \begin{array}{ll} 1 &{} \text { if } s_{2}^{d}\ge r3 \\ 0 &{} \text { else } \end{array}\right. } \end{aligned}$$

12 $$\begin{aligned} bstep_{3}^{d}= & {} {\left\{ \begin{array}{ll} 1 &{} \text { if } s_{3}^{d}\ge r3 \\ 0 &{} \text { else } \end{array}\right. } \end{aligned}$$

The distances that *i* will move relative to $\alpha$, $\beta$, and $\delta$ are known as $bstep_{1}$, $bstep_{2}$, and $bstep_{3}$. $X_1$, $X_2$, and $X_3$, which are calculated in Eqs. (13), (14), and (15), are binary vectors affected by the movement of $\alpha$, $\beta$ and $\delta$ wolves, respectively.

13 $$\begin{aligned} X_{1}^{d}= & {} {\left\{ \begin{array}{ll} 1 &{} \text { if } X_{\alpha }^{d}+bstep_{1}^{d}\ge 1 \\ 0 &{} \text { else } \end{array}\right. } \end{aligned}$$

14 $$\begin{aligned} X_{2}^{d}= & {} {\left\{ \begin{array}{ll} 1 &{} \text { if } X_{\beta }^{d}+bstep_{2}^{d}\ge 1 \\ 0 &{} \text { else } \end{array}\right. } \end{aligned}$$

15 $$\begin{aligned} X_{3}^{d}= & {} {\left\{ \begin{array}{ll} 1 &{} \text { if } X_{\delta }^{d}+bstep_{3}^{d}\ge 1 \\ 0 &{} \text { else } \end{array}\right. } \end{aligned}$$

After obtaining $X_{1}^{d}$, $X_{2}^{d}$, and $X_{3}^{d}$, the position of $X_i$ in the next iteration ($t+1$) is updated using a simple stochastic crossover as Eq. (16). Where *r*4 represents a random number chosen from the uniform distribution $\in [0, 1]$

16 $$\begin{aligned} X_{i}^{d}(t+1)={\left\{ \begin{array}{ll} X_{1}^{d} &{} \text { if } r4< \frac{1}{3} \\ X_{2}^{d} &{} \text { elseif } \frac{1}{3} \le r4 < \frac{2}{3}\\ X_{3}^{d} &{} \text { else } \end{array}\right. } \end{aligned}$$

**Algorithm 1:**
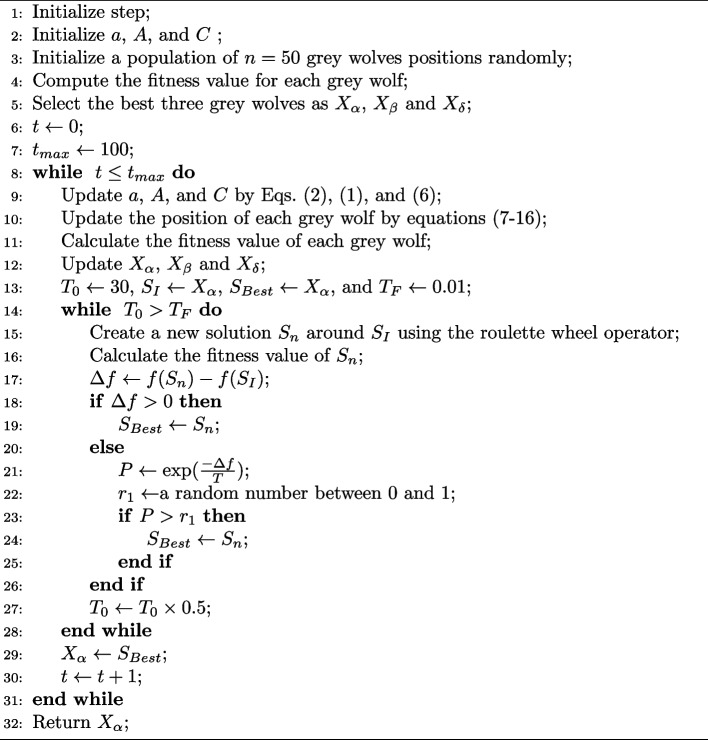
Discrete Grey Wolf Optimizer (BGWO)

#### SA

SA is an optimization algorithm based on annealing that mimics how the material is heated and slowly cooled to achieve a uniform structure [49]. The algorithm generates an initial random solution. Then, in subsequent iterations, a new neighborhood solution is generated and evaluated using the fitness function. Whenever a new solution is better, it is accepted, whereas when it is the worst, it is accepted based on a Boltzmann probability.

17 $$\begin{aligned} P=\exp \left( \frac{-\Delta {f}}{T}\right) \end{aligned}$$

In Eq. (17), $\Delta {f}$ represents the difference between the previous and new solutions’ fitness values, and T represents the temperature parameter.

At the end of each iteration of the grey wolf algorithm, we enter the value of $X_\alpha$ as the initial value into the SA algorithm and select a neighborhood solution using the roulette wheel strategy. To generate the neighborhood solution, the roulette wheel operator chooses one of the Swap, Insertion, Inversion, R2L (Rotate to Left), or R2R (Rotate to Right) operators. After a neighborhood is created, its fitness value is measured and compared with the fitness value of the input $X_\alpha$. If we conclude that the neighbor solution is accepted due to the higher fitness value or by using the Boltzmann probability, we will replace it with the initial value. Otherwise, we will create another neighborhood until we reach the predetermined temperature. Therefore, we go to the next step of the grey wolf and use the new value of $X_\alpha$. This process is shown in pseudocode 1.

#### Objective function

Our methodology incorporates an ensemble learning approach, utilizing four distinct ML algorithms, XGBoost ($clf_{1}$), SVM ($clf_{2}$), RF ($clf_{3}$), and DT ($clf_{4}$), to determine the fitness of potential solutions.

We commence by partitioning our merged dataset into three subsets: 60% for training, 20% for validation, and the remaining 20% for testing. The process initiates with selecting the features present in the potential solution, followed by training the ensemble using 60% of the samples from the training set. We implemented Synthetic Minority Over-sampling Technique (SMOTE) on the training set to address class imbalance, while the validation and test sets were kept in their original, untouched state.

Posttraining, we employ the validation set to assess the performance of the base classifiers. This evaluation paves the way for the computation of their respective weights, followed by a weighted voting procedure to derive the results of the ensemble strategy. This entire workflow is meticulously repeated as necessary to compute the fitness value for varying solutions, as guided by Eqs. (18) and (19). In addition, the final 20% of the dataset, designated as the testing set, is utilized for gauging the model’s performance and facilitating comparative analyses.

18 $$\begin{aligned} Avg_{Eclf}= & {} \frac{Ac_{Eclf} + Pr_{Eclf} + Re_{Eclf} + F1_{Eclf} + F2_{Eclf}}{5} \end{aligned}$$

19 $$\begin{aligned} Fit(Sol)= & {} (\varphi \times Avg_{Eclf} ) + \left( (1-\varphi ) \times \frac{NF}{AF}\right) \end{aligned}$$

In the above equations, the fitness value of a solution, denoted as *Fit*(*Sol*), is computed by averaging the evaluation metrics of the Ensemble classifier (*Eclf*), with $\varphi = 0.8$ representing the significance attached to the number of selected features. Here, *NF* is the number of selected features, and *AF* is the total number of features in the dataset.

The symbols *Ac*, *Pr*, *Re*, *F*1, and *F*2 represent the evaluation metrics: Accuracy, Precision, Recall, F1 score, and F2 score, respectively. These metrics are detailed in Eqs. (20), (21), (22), (23), and (24).

20 $$\begin{aligned} Ac= & {} \frac{TP+TN}{TP+FP+FN+TN} \end{aligned}$$

21 $$\begin{aligned} Pr= & {} \frac{TP}{TP+FP} \end{aligned}$$

22 $$\begin{aligned} Re= & {} \frac{TP}{TP+FN} \end{aligned}$$

23 $$\begin{aligned} F1= & {} 2 \times \left( \frac{Pr \times Re}{Pr + Re}\right) \end{aligned}$$

24 $$\begin{aligned} F2= & {} \frac{5 \times Pr \times Re}{(4 \times Pr) + Re} \end{aligned}$$

Given that cancer samples are considered positive and normal samples are considered negative, the values of TP, TN, FP, and FN in the above equations are defined as follows:

- *True Positive (TP)* Correctly predicted cancer instances.
- *True Negative (TN)* Correctly predicted normal instances.
- *False Positive (FP)* Normal instances wrongly predicted as cancer.
- *False Negative (FN)* Cancer instances wrongly predicted as normal.

To implement weighted voting, we calculate a weight for each base classifier post-training, utilizing the validation set, as depicted in Eq. 25.

25 $$\begin{aligned} fw_{clf_{i}}= \frac{Ac_{clf_{i}} \times Pr_{clf_{i}} \times Re_{clf_{i}} \times F1_{clf_{i}} \times F2_{clf_{i}}}{\sum _{k=1}^{4} (Ac_{clf_{k}} \times Pr_{clf_{k}} \times Re_{clf_{k}} \times F1_{clf_{k}} \times F2_{clf_{k}})} \end{aligned}$$

In the weighted voting procedure, we amalgamate the results from each base classifier employing their respective weights, $fw_{clf_{i}}$, which ensures that classifiers demonstrating superior performance on the validation set exercise a more substantial influence on the ensemble’s final prediction.

The ensemble classifier’s prediction for each sample can be expressed as:

26 $$\begin{aligned} Pred_{Eclf}(x) = \arg \max _{c \in C} \sum _{i=1}^{4} fw_{clf_{i}} \times Pred_{clf_{i}}(x) \end{aligned}$$

In Eq. (26), $Pred_{Eclf}(x)$ is the class predicted for sample *x* by the ensemble classifier, *C* is the set of possible class labels, and $Pred_{clf_{i}}(x)$ is the class predicted for sample *x* by base classifier $clf_{i}$.

The performance metrics $Ac_{Eclf}$, $Pr_{Eclf}$, $Re_{Eclf}$, $F1_{Eclf}$, and $F2_{Eclf}$ of the ensemble classifier are then computed using the predictions from the ensemble classifier *Eclf*, as defined in Eqs. (20), (21), (22), (23), and (24).

Importantly, the ensemble’s performance typically surpasses the performance of its individual components, especially when the base classifiers are diverse. This is because the ensemble leverages each classifier’s strengths while mitigating their weaknesses.

### BGWO_Ens, GA_Ens, and LASSO feature selection algorithms

#### BGWO_Ens

BGWO_Ens is akin to BGWO_SA_Ens, with the primary distinction being the absence of the SA process. It harnesses the BGWO algorithm, which is a variant of the GWO tailored for binary-encoded problems, making it suitable for FS. After determining the optimal subset of features using BGWO, these selected features are fed into an ensemble of classifiers, structured similarly to the ensemble method delineated in section “Objective function”. By integrating the search capabilities of BGWO with the predictive power of ensemble models, this method aims to achieve high predictive accuracy while minimizing overfitting, particularly in high-dimensional datasets.

#### GA_Ens

The GA_Ens method synergizes GA with ensemble learning. Genetic Algorithms are heuristic search algorithms inspired by natural selection. Potential feature subsets are encoded as chromosomes, which evolve over generations to identify the most effective subsets. Once the optimal features are selected using GA, they are then used to train an ensemble of ML models, following the ensemble structure detailed in section “Objective function”. The melding of GA’s evolutionary search with ensemble modeling offers a robust method that adeptly handles FS and prediction tasks.

#### LASSO

LASSO (Least Absolute Shrinkage and Selection Operator) is a regression-based method incorporating L1 regularization. This regularization can push some feature coefficients to be precisely zero, allowing LASSO to inherently perform FS by excluding features with zero coefficients. Unlike BGWO_Ens and GA_Ens, LASSO does not naturally integrate an ensemble approach. However, its prowess lies in its ability to manage multicollinearity, select a feature subset while concurrently performing regression, and offer ease of interpretation.

To remove the repeated information and streamline the content in the two sections, here is a revised version:

#### MCFS-IFS

In this study, the Monte Carlo Feature Selection (MCFS) method [48] was employed for feature selection from gene expression datasets. The datasets were prepared and divided into training and test sets, with the training set balanced using the SMOTE. The MCFS process, conducted over 100 iterations, involved selecting and evaluating a random subset of 5000 features from the training dataset in each iteration. These features were assessed using a RF Classifier and a 10-fold cross-validation approach. The key aspect was scoring each feature based on its presence in high-performing subsets, thereby determining its importance in predicting the target variable. Post-iteration, the top 5000 features were ranked according to their scores for further analysis.

Incremental Feature Selection (IFS) [50] was then applied to refine this feature set, involving iterative evaluation of feature subsets, beginning with the top 5 features and increasing in size by 5 features at each step. A range of classifiers, including DT, KNN, RF, SVM, and NN, were used to assess the performance of each subset using 10-fold cross-validation. Various metrics such as F1 score, PR-AUC, ROC-AUC, Mathews correlation coeffcient (MCC), and Balanced Accuracy (BAc) where MCC and BAc can be computed by Eqs. (27) and (28) were employed for a comprehensive assessment. The subset that achieved the highest F1 score was identified as the optimal feature set and was further tested on a separate test set to validate its generalizability and effectiveness.

27 $$\begin{aligned} MCC= & {} {\frac{TP\times TN-FP\times FN}{\sqrt{(TP+FP)(TP+FN)(TN+FP)(TN+FN)}}} \end{aligned}$$

28 $$\begin{aligned} BAc= & {} \frac{1}{2}\left( \frac{TP}{TP+FN}+\frac{TN}{TN+FP}\right) \end{aligned}$$

#### mRMR-IFS

Similarly, the mRMR (Minimum redundancy maximum relevance) feature selection method [51]was integrated with IFS to enhance the robustness and accuracy of our gene expression data analysis in BC research. Using the ‘pymrmr‘ Python library, the mRMR algorithm selected the top 5000 features from over 10,000 potential genes based on their mutual information quotient with the target variable. This selection ensured that each feature contributed uniquely to the predictive model.

The subsequent IFS process evaluated these mRMR-selected features in smaller subsets to determine the most impactful set for predictive modeling. Starting with the top 5 features and incrementally increasing by 5 in each iteration, the process assessed feature subsets up to the top 5000. Performance evaluation through a 10-fold cross-validation with classifiers like DT, KNN, RF, and SVM, along with metrics including F1 score, ROC-AUC, PR-AUC, MCC, and BAc, helped identify the best feature set. This rigorous approach pinpointed the most influential genes for breast cancer detection and diagnosis, significantly contributing to the advancement of breast cancer biomarker discovery.

### Superior genes selection

In the intricate landscape of genomics, different analytical methods can yield varying gene sets of interest. Our approach involved conducting Differential Expression (DE) analysis and the BGWO_SA_Ens feature selection method as two independent processes across two distinct datasets. While DE analysis identifies genes that manifest a significant change in expression levels between conditions, the BGWO_SA_Ens feature selection is applied to assess that the genes are also impactful in predictive modeling. As a result, the intersection of these methods (two sets from DE analysis and two sets from BGWO_SA_Ens across the datasets) provides us with a set of “superior genes” that are both differentially expressed and integral in ML predictions. This dual analysis approach allows for a comprehensive and unbiased identification of key genes, ensuring a robust selection of candidates for further biological investigation.

To further elucidate the roles and implications of these superior genes, three advanced analyses were undertaken. First, an enrichment analysis was performed to uncover the underlying biological pathways and processes in which these genes are predominantly involved, providing insights into their functional significance. Subsequently, network analysis furnished a clearer picture of the interrelations and interactions among these genes, offering a systems biology perspective on their collective behavior and potential regulatory patterns. Last, by investigating potential drug interactions, we probed the therapeutic avenues and implications these genes might open up, potentially paving the way for targeted treatments or pharmacological interventions. Collectively, these analyses not only amplify our understanding of the identified superior genes but also fortify their relevance in both research and therapeutic contexts.

### Enrichment analysis of superior genes

Enrichment analysis deciphers the functional roles of superior genes highlighted through DE analysis and ML. By statistically assessing the association between these genes and specific diseases, the analysis furnishes gene-set profiles. This finding sheds light on the involvement of these genes in cancer progression. KEGG pathway enrichment analysis and GO [52, 53] further enhance this understanding, offering classifications such as molecular functions (MF), biological processes (BP), and cellular components (CC).

### PPI network analysis

Analyzing protein-protein interactions (PPI) bridges the knowledge gap between superior genes and cellular functionality [54]. Utilizing the STRING database [55], we mapped interactions with a confidence threshold of 0.4, visualized through Cytoscape 3.9.1. Key regions in this PPI network were pinpointed using the MCODE plugin, while the cytoHubba plugin highlighted influential genes within the network. This holistic approach unravels the intricacies of gene interactions, potentially directing us towards novel therapeutic avenues.

### Exploration of drug interactions with superior genes

Understanding drug-gene dynamics is pivotal for crafting effective BC treatments. Researchers can sculpt more individualized, effective therapeutic strategies by studying the interplay between drugs and disease-related genes. The DGIdb database [56] served as a resource for unearthing drugs that potentially interact with our superior genes. Visual representations, crafted using Cytoscape, further clarify these interactions, propelling insights into disease biology and therapeutic innovation.

## Results

### Data preprocessing and merging

Following the meticulous preprocessing of the GSE10810, GSE42568, and GSE45827 datasets, refined gene expression matrices were obtained for each of these datasets. This processing consisted of 179 samples in the merged dataset and 155 samples in the GSE45827 dataset, encompassing 135 BC samples and 44 normal samples in the merged dataset, and 144 BC samples with 11 normal samples in the GSE45827 dataset. The preprocessing steps resulted in a comprehensive set of 10,629 genes for analysis in the merged dataset, and 11,731 genes in the GSE45827 dataset.

**Fig. 1:**
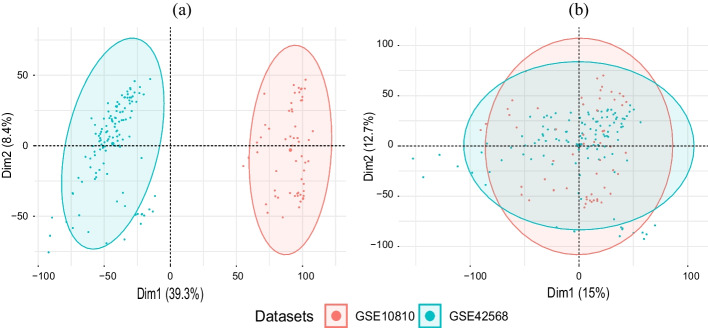
PCA visualization of the merged dataset **a** before and **b** after batch effect removal using the ComBat function

After the preprocessing phase, a PCA plot was generated to visualize the batch effects inherent in the merged dataset. The evident clustering of samples from different datasets illustrated the presence of these batch effects as shown in Fig. 1a. However, after applying the empirical Bayes algorithm through the ComBat function, a subsequent PCA plot showcased the mitigation of these batch effects. The samples were no longer clustered based on their source dataset, as shown in Fig. 1b, which indicated the successful removal of the batch effects.

**Fig. 2:**
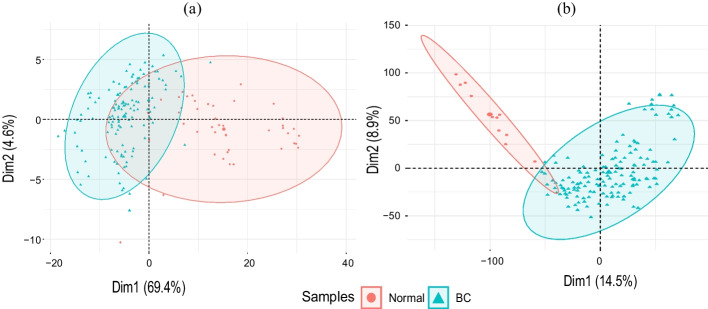
PCA plots of BC and normal samples for **a** merged dataset and **b** GSE45827 dataset

Additionally, PCA plots elucidating the clustering of samples were constructed as displayed in Fig. 2. BC samples distinctly grouped together, apart from the normal samples, revealing the inherent differences in their gene expression patterns. This demarcation in the PCA space affirms the significance of the gene expression features in distinguishing between BC and normal samples in both the merged dataset 2a and the GSE45827 dataset 2b.

### Differentially expressed genes identification

From the comprehensive analysis of the 10,629 genes in the merged dataset, 164 genes were discerned as differentially expressed. These were delineated based on the following criteria: Adjusted *p*-value < 0.05 and $|log_{2} \;{fold change (FC)}|> 2$. Consequently, from the identified DEGs, 34 genes exhibited upregulation while 130 genes were found to be downregulated. Additionally, analysis of the GSE45827 dataset revealed 350 DEGs among 11731 genes, with 208 upregulated and 142 downregulated genes. Table 1 summarizes the count of genes after DEG analysis for both datasets.

**Table 1:** Quantitative distribution of differentially expressed genes, upregulated and downregulated entities

Dataset	Total genes	DEGs	Upregulated	Downregulated
Merged Dataset	10629	164	34	130
GSE 45827	11731	350	208	142

**Fig. 3:**
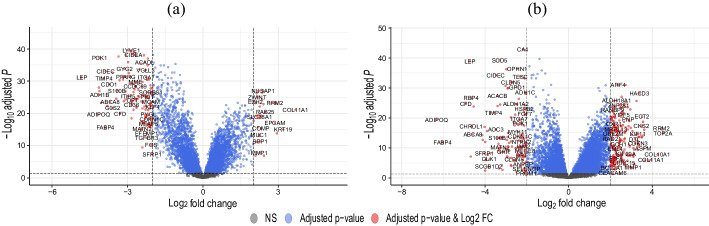
Graphical Representation of DEGs via Volcano Plot for **a** merged dataset and **b** GSE45827 dataset

These identified DEGs significantly contributed to the statistical demarcation between the BC and normal samples within the merged dataset, as well as the GSE 45827 dataset. Figure 3 illustrates the volcano plot of these DEGs. The plot’s X-axis signifies the fold change, while the Y-axis represents the $log_{10}$ transformed Adjusted *p*-value for every gene. The DEGs are prominently marked in red and annotated accordingly.

**Fig. 4:**
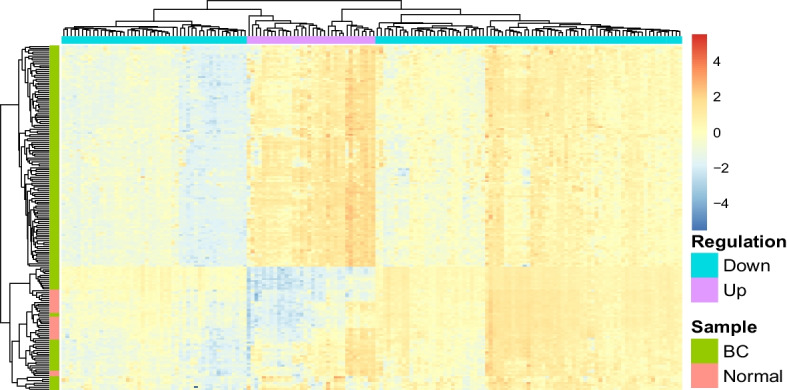
Clustering heatmap for the merged dataset distinguishing BC from normal profiles

**Fig. 5:**
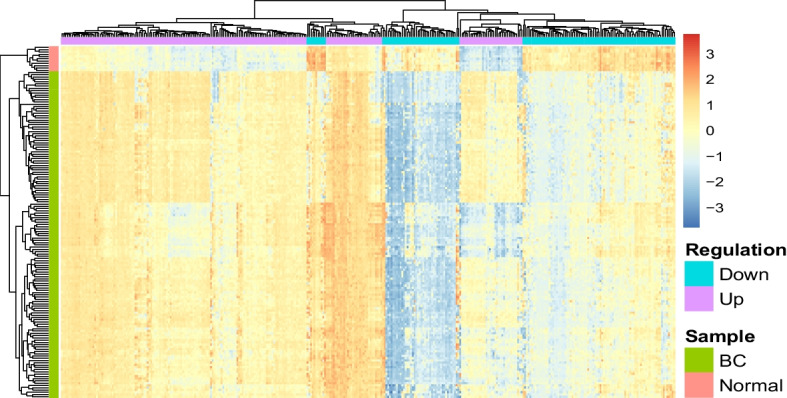
Clustering heatmap for the GSE45827 dataset distinguishing BC from normal profiles

Furthermore, heatmaps were generated to provide a visual representation of the distinction between samples based on their group classification as illustrated in Figs. 4 and 5. The hierarchical clustering depicted in each heatmap efficiently segregated the BC samples from the normal samples, emphasizing the effectiveness of the selected gene expression features in discriminating between the two groups. Notably, the gradient in the heatmaps, marked by distinct color patterns for BC and normal samples, underlines the pronounced differences in their gene expression profiles for both the merged dataset and the GSE45827 dataset.

### Feature selection results

#### BGWO_SA_Ens performance

We executed the BGWO_SA_Ens method ten times, yielding ten different feature sets, designated Fset1 through Fset10, for each dataset. Each of these feature sets was then used to test six base classifiers, XGBoost, DT, RF, SVM, KNN, and NN, using a test set that only contained features selected by BGWO_SA_Ens. This was carried out for all ten selected feature sets for each dataset. Subsequently, we computed the F1, PR-AUC, and ROC-AUC evaluation metrics to assess the performance of the selected features. The results, reflecting the mean values derived from the six base classifier methods, are presented below.

**Table 2:** Performance metrics for each feature set obtained using BGWO_SA_Ens in Merged Dataset and GSE45827

Fset	Dataset	NF	FV $^{1}$	F1	PR-AUC	ROC-AUC
Fset1	Merged Dataset	1610	0.059	0.942	0.984	0.953
Fset2	Merged Dataset	2027	0.063	0.936	0.981	0.946
Fset3	Merged Dataset	1864	0.059	0.931	0.964	0.873
Fset4	Merged Dataset	3055	0.072	0.791	0.981	0.955
Fset5	Merged Dataset	1900	0.058	0.931	0.963	0.872
Fset6	Merged Dataset	3108	0.064	0.794	0.978	0.938
**Fset7** $^{2}$	**Merged Dataset**	**1404**	**0.044**	**0.981**	**0.998**	**0.995**
Fset8	Merged Dataset	3255	0.061	0.791	0.967	0.879
Fset9	Merged Dataset	1763	0.051	0.769	0.958	0.857
Fset10	Merged Dataset	3134	0.061	0.791	0.980	0.948
Fset1	GSE45827	3018	0.059	0.910	0.978	0.956
**Fset2** $^{2}$	**GSE45827**	**1710**	**0.046**	**0.965**	**0.986**	**0.972**
Fset3	GSE45827	2100	0.061	0.914	0.968	0.939
Fset4	GSE45827	3914	0.063	0.879	0.969	0.954
Fset5	GSE45827	1636	0.051	0.901	0.976	0.959
Fset6	GSE45827	2126	0.047	0.931	0.977	0.956
Fset7	GSE45827	3222	0.061	0.910	0.973	0.948
Fset8	GSE45827	2219	0.047	0.936	0.978	0.956
Fset9	GSE45827	2670	0.049	0.958	0.977	0.955
Fset10	GSE45827	3899	0.060	0.892	0.973	0.947

$$^{1}$$ Fitness Value

$^{2}$ Best performing feature set for the dataset

**Fig. 6:**
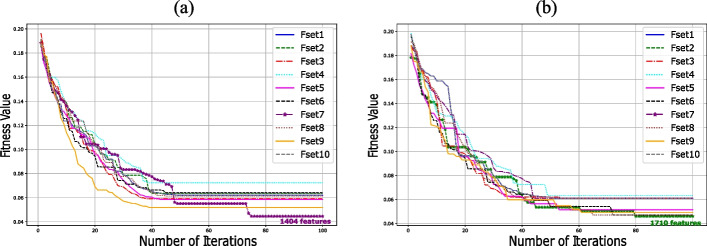
Comparative Analysis of Feature Selection Convergence: **a** Merged Dataset and **b** GSE45827 Dataset using BGWO_SA_Ens Method

**Fig. 7:**
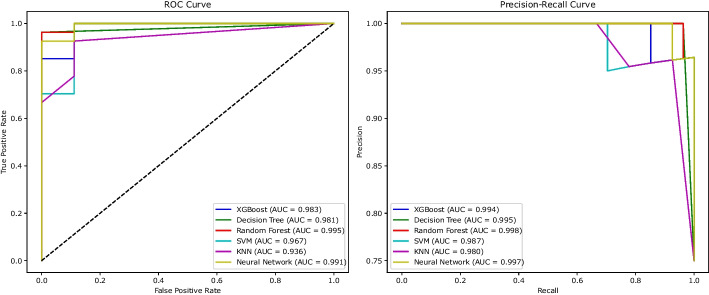
ROC-AUC and PR-AUC performance of Fset7 in the Merged Dataset

**Fig. 8:**
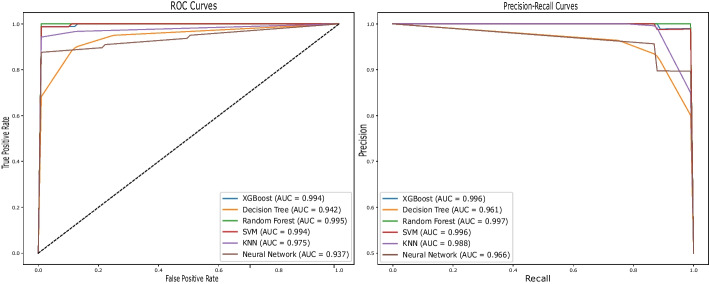
ROC-AUC and PR-AUC performance of Fset2 in the GSE45827 Dataset

From Table 2, it is evident that Fset7 exhibited superior performance compared to the other sets in the Merged Dataset, and Fset2 was the best performer in the GSE45827 dataset, as indicated by the rows in bold text. These feature sets encompassed a selection of 1404 genes for the merged dataset and 1710 genes for the GSE45827 dataset. Figures 6a and 6b display the optimization plots for the merged dataset and the GSE45827 dataset, respectively. In Fig. 6a, it is demonstrated that Fset7 attained a better fitness value (FV) than other feature sets at iteration 100 for the merged dataset. Similarly, Fig. 6b shows that Fset2 outperformed other feature sets at iteration 100 in the GSE45827 dataset. Additionally, Figures 7 and 8 illustrate the PR-AUC and ROC-AUC performance of Fset7 in the merged dataset and Fset2 in the GSE45827 dataset, respectively.

#### Comparison to other methods (BGWO_Ens, GA_Ens, LASSO, MCFS-IFS, mRMR-IFS)

In our comprehensive analysis, we conducted a comparative evaluation of various FS techniques, including BGWO_Ens, GA_Ens, LASSO, MCFS-IFS, mRMR-IFS,applied to both the merged dataset and the GSE45827 dataset for breast cancer biomarker identification. These FS methods yielded significantly different sets of genes, with BGWO_Ens identifying 1941 in the merged dataset and 2114 in the GSE45827 dataset, GA_Ens 2557 in the merged dataset and 2488 in the GSE45827 dataset, LASSO 10 in the merged dataset and 8 in the GSE45827 dataset, MCFS-IFS 1850 in the merged dataset and 475 in the GSE45827 dataset, and mRMR-IFS 355 in the merged dataset and 1660 in the GSE45827 dataset, indicating a wide variance in feature selection granularity.

To assess the performance and effectiveness of these FS strategies, we incorporated the selected features within our test dataset. We proceeded to employ six distinct base classifiers, XGBoost, DT, RF, SVM, KNN, and NN, to evaluate the predictive power of the features identified by each FS method. Classifiers were rigorously tested using 10-fold cross-validation to ensure stability and reliability in the feature evaluation process. The final feature set was determined for each FS technique, comparable to the best feature set (Fset) identified by our BGWO_SA_Ens method.

The results are meticulously summarized in Table 3, presenting a side-by-side comparison of the most effective outcomes derived from the five FS techniques. In this table, the bold text highlights the best performing algorithm that most accurately predict BC, as evidenced by the metrics given. This comparative analysis extends to the results obtained from the features selected by BGWO_SA_Ens and contrasts them against the performance when the full spectrum of features is employed. Notably, the ROC and Precision-Recall curves, as illustrated in the Figs. 7 and 8, provide a visual and quantitative representation of the classifiers’ performance, with AUC scores serving as a benchmark for comparison.

**Table 3:** Comparison of the performance metrics of different feature selection methods

Algorithm	Dataset	NF	F1	PR-AUC	ROC-AUC	MCC	BAc
All Features	Merged Dataset	10629	0.862	0.870	0.858	0.814	0.905
	GSE45827	11731	0.873	.978	0.961	0.895	0.795
**BGWO_SA_Ens**	Merged Dataset	1404	0.984	0.986	0.977	0.967	0.977
	GSE45827	1710	0.965	0.986	0.972	0.953	0.965
BGWO_Ens	Merged Dataset	1941	0.898	0.972	0.965	0.839	0.916
	GSE45827	2114	0.865	0.965	0.931	0.829	0.914
GA_Ens	Merged Dataset	2557	0.954	0.986	0.977	0.943	0.965
	GSE45827	2488	0.908	0.986	0.972	0.894	0.947
LASSO	Merged Dataset	10	0.885	0.874	0.852	0.831	0.913
	GSE45827	8	0.729	0.904	0.872	0.571	0.772
MCFS-IFS	Merged Dataset	1850	0.940	0.979	0.973	0.945	0.898
	GSE45827	475	0.951	0.975	0.963	0.930	0.866
mRMR-IFS	Merged Dataset	355	0.945	0.971	0.954	0.916	0.953
	GSE45827	1660	0.926	0.940	0.964	0.897	0.934

The Random Forest classifier, for instance, achieved AUC scores of 0.995 in ROC and 0.998 in Precision-Recall metrics for the merged dataset, and similarly, 0.995 in ROC and 0.998 in Precision-Recall metrics for the GSE45827 dataset. These results indicate exceptional classification accuracy. Such consistency across different datasets highlights the potential of our hybrid BGWO_SA_Ens approach in identifying the most predictive biomarkers for BC, potentially paving the way for more targeted and effective therapeutic strategies.

### Superior genes identification

In our analysisacross two distinct datasets, we identified different sets of differentially expressed genes (DEGs) and selected genes using the BGWO_SA_Ens algorithm. For the merged dataset, a total of 164 genes were identified as DEGs, and the BGWO_SA_Ens algorithm selected 1404 genes. In contrast, for the GSE45827 dataset, 350 genes were identified as DEGs, with the BGWO_SA_Ens algorithm selecting 1710 genes. An intersection of these four sets (DEGs and BGWO_SA_Ens selected genes from both datasets) revealed a commonality of 35 genes. These overlapping genes, which are present in both the DEGs and the BGWO_SA_Ens selected genes from each dataset, are referred to as the “superior genes.” This subset represents genes that are consistently significant across different analytical methods and datasets, highlighting their potential importance in the context of breast cancer.

**Fig. 9:**
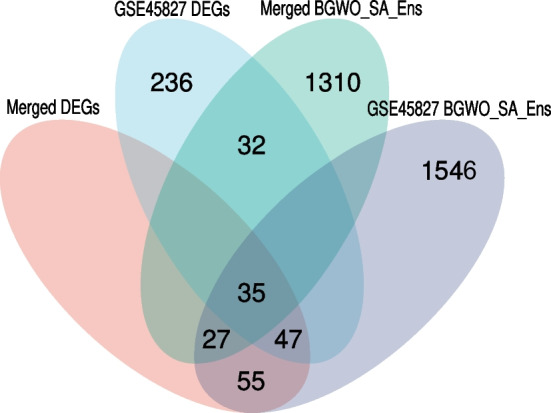
Venn diagram illustrating the intersection of DEGs and BGWO_SA_Ens selected genes. The overlapping region denotes the identified superior genes. Venn diagram illustrating the intersection of DEGs and BGWO_SA_Ens selected genes from the merged and GSE45827 datasets. The overlapping region denotes the identified superior genes

The relationship and overlap between the DEGs and the genes selected through the BGWO_SA_Ens algorithm across both datasets are schematically represented in Fig. 9, a Venn diagram. This visualization emphasizes the significance of the identified superior genes and provides a clear depiction of their derivation from the intersection of the two methodologies and datasets.

### Enrichment analysis results

The comprehensive enrichment analysis was segmented into two pivotal sections, focusing on GO enrichment and KEGG pathway enrichment respectively.

#### GO enrichment

**Fig. 10:**
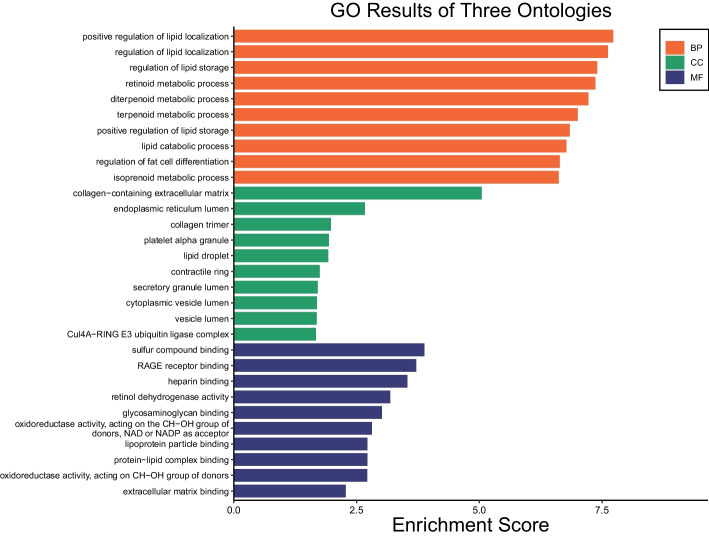
GO enrichment analysis results categorized into MF, BP, and CC. The EnrichmentScore demonstrates the importance of each category in relation to the 35 superior genes. Distinct color codings for MF, BP, and CC allow for easy differentiation and visualization of the results

Using GO analysis, the molecular characteristics of the 35 superior genes were delineated across three ontological categories. In terms of MF, the most significant functionalities identified were *sulfur compound binding*, *RAGE receptor binding*, and *heparin binding*, each possessing a noteworthy enrichment score. Within the BP category, processes such as *positive regulation of lipid localization*, *regulation of lipid localization*, and *regulation of lipid storage* were prominently enriched. In the context of CCs, the key enriched components comprised *collagen containing extracellular matrix*, *endoplasmic reticulum lumen*, and *collagen trimer*. The significance of these functionalities, processes, and components is underscored by their respective Enrichment Scores, highlighting their potential pivotal roles in the physiological manifestations observed. The comprehensive visual representation of these GO enrichments is illustrated in Fig. 10.

#### KEGG pathway enrichment

**Fig. 11:**
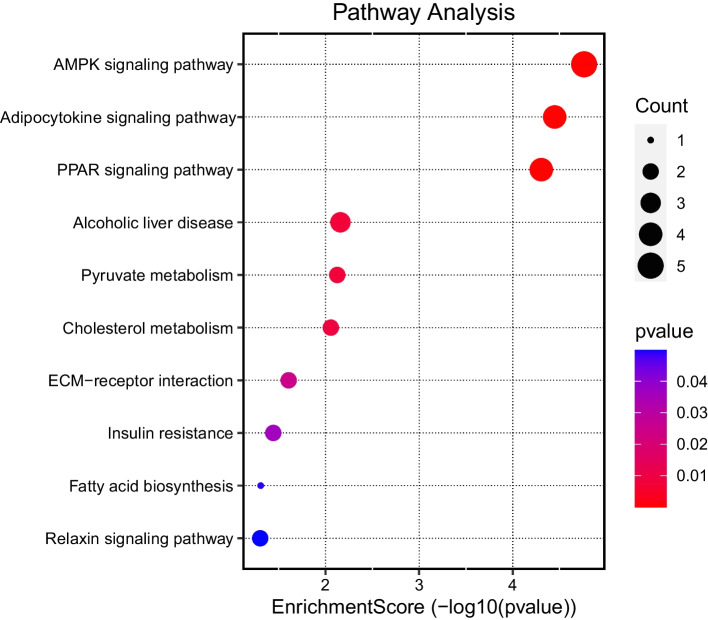
KEGG pathway analysis displaying the enrichment score of various pathways related to the 35 superior genes. The pathways shown are ranked based on their significance and relevance. A higher EnrichmentScore indicates greater significance. The count and *p*-value further provide insight into the number of genes involved and the statistical significance of each pathway, respectively

The KEGG analysis spotlighted several pathways potentially intertwined with the biological activities of the superior genes. Preeminent among these pathways were the *AMPK signaling pathway*, *Adipocytokine signaling pathway*, and *PPAR signaling pathway*. The *AMPK signaling pathway*, with the highest EnrichmentScore, is particularly pivotal, suggesting a potentially significant involvement in the processes under investigation. Other pathways such as *Alcoholic liver disease* and *Pyruvate metabolism* also exhibited consequential EnrichmentScores, indicative of their importance in the broader molecular schema. A comprehensive visual overview of the KEGG pathway enrichments is presented in Fig. 11.

### Analysis of protein-protein interaction networks

Within this section, we probe the nuanced realm of protein-protein interactions (PPI) pertaining to our designated cohort of superior genes. Our objective is to elucidate the concealed links and underscores pivotal subnetworks that might elucidate BC at a molecular level.

**Fig. 12:**
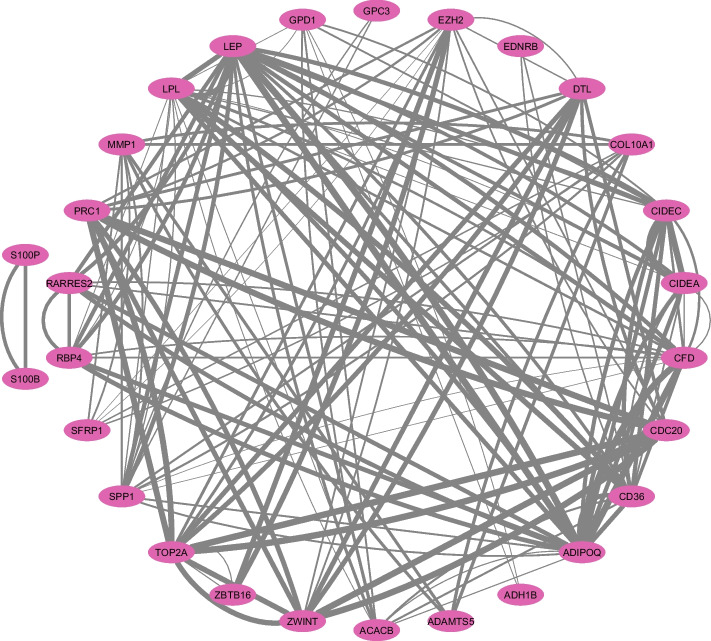
Integrated Protein-Protein Interaction (PPI) network schematic of 28 superior genes, representing a subset of the total 35 identified superior genes with known interactions. The thickness of the lines between nodes corresponds to their combined score

As illustrated in Fig. 12, the PPI network manifests as a complex matrix of interactions encompassing the delineated superior genes. Individual nodes in this construct signify the superior genes, and the connecting edges depict the probable affiliations and mutual interactions between them. This all-encompassing illustration provides a succinct perspective on the molecular dynamics inherent to BC.

**Fig. 13:**
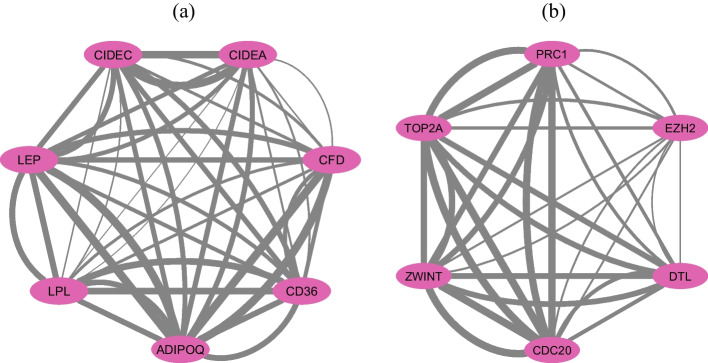
Principal subnetworks: **a** MCODE metric of 7 **b** MCODE metric of 6. The thickness of the lines between nodes corresponds to their combined score

Embedded within this multifaceted PPI network, the MCODE tool has identified regions with high connectivity, illustrated as subnetworks in Figure 13. Such subnetworks typically signify proteins that synergistically partake in analogous biological functionalities or pathways. They possess the capability to unveil seminal insights into BC’s molecular mechanics.

The inaugural subnetwork, depicted in Fig. 13a, is conferred an MCODE metric of 7, insinuating elevated interconnectivity among its constituent proteins. This conglomerate potentially typifies a cadre of proteins synergistically operating within a distinct cellular trajectory, thereby hinting at their implications in BC molecular dynamics.

The sequential subnetwork, represented in Fig. 13b, while being less expansive and having diminished connectivity in comparison to the former, remain of paramount relevance. The second registers an MCODE metric of 6, insinuating its representation of separate functional assemblies within BC’s multifarious molecular puzzle.

To discern the paramount nodes within these subnetworks, our approach was augmented by the cytoHubba tool. Nodes characterized by elevated centrality metrics occupy cardinal positions within the construct, potentially modulating its overarching framework and operational dynamics. Such quintessential proteins might exert pivotal functions in the pathogenesis, rendering them prospective foci for therapeutic endeavors.

These revelations furnish profound insights into the intricate molecular orchestration linked to BC. By emphasizing these superior genes and their interrelationships, potential avenues for therapeutic strategies may surface. Further experimental verification of these networks and their pivotal nodes will strengthen their relevance in BC and could accelerate the development of innovative therapeutic approaches.

### Investigation of drug-gene interactions in oncology

Delineating the nuanced interactions between pharmacological agents and genetic elements in BC is crucial for the evolution of therapeutic modalities. The discernment and systematic study of these drug-gene interactions pave the way for the formulation of targeted therapeutic regimens, enhancement of treatment precision, reduction of adverse reactions, and the optimization of patient prognosis. The prominence of genes in oncogenic predisposition and disease trajectory emphasizes the imperative of comprehending the molecular dynamics between drugs and the specific genes implicated in oncogenesis.

For this study, we leveraged the DGIdb (v4.2.0) database, an exhaustive repository encompassing drug-gene associations and potential drug target genes. This database integrates data spanning diverse origins, including peer-reviewed literature, solidifying its authority on drug-gene synergies.

**Fig. 14:**
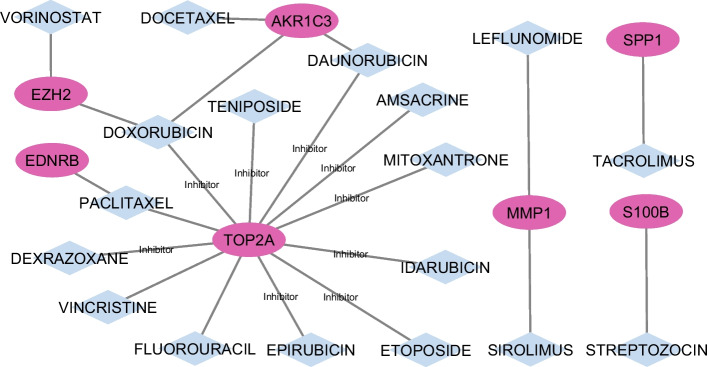
Graphical elucidation of drug-gene dynamics, highlighting the multifaceted therapeutic frontier

To decipher and represent the aforementioned drug-gene associations, we employed Cytoscape, a sophisticated tool dedicated to network synthesis and visualization. Figure 14 showcases the derived network, with pharmacological agents delineated as blue nodes and genetic entities as pink nodes. This graphical abstraction elucidates the dense meshwork of interactions between pivotal genes and their corresponding pharmacological modulators. Importantly, this networked representation imparts a nuanced view of associations that might remain obscured in a mere tabulated format.

To encapsulate, the drug-gene interconnection illustrated in Fig. 14 provides invaluable insight into the labyrinthine domain of drug-gene dynamics. Such a perspective augments our comprehension of prospective therapeutic trajectories and propels the formulation of bespoke therapeutic solutions for BC, epitomizing the potential of precision medicine within the realm of oncology.

## Discussion

The genetic intricacies of BC call for advanced methods in biomarker discovery. Confronting the high-dimensionality of gene expression data, our BGWO_SA_Ens algorithm effectively discerns crucial biomarkers indicative of BC. We addressed the constraints of sample size by merging datasets, thus broadening the scope of our meta-analysis.

The incorporation of ensemble learning, which leverages multiple classifiers, is pivotal to the BGWO_SA_Ens algorithm’s success in honing gene subset selection. The adeptness of ensemble learning in managing complex data bolsters the feature selection strength of BGWO_SA_Ens. It results in more accurate predictions and versatile model applications. Moreover, the combination of BGWO and SA’s FS capabilities ensures robust and reliable results.

While BGWO_SA_Ens, BGWO_Ens, and GA_Ens represent advanced metaheuristic algorithms that leverage the strengths of ML to find optimal solutions, their comparison with methods like LASSO, MCFS-IFS, and mRMR-IFS offers a broader perspective on FS approaches. LASSO, known for its simplicity and effectiveness in feature reduction, tends to select a smaller set of features but may oversimplify complex biological data, as indicated by its lower performance metrics in our study. On the other hand, MCFS-IFS and mRMR-IFS, while offering more comprehensive feature selection, may not always provide the optimal balance between feature reduction and predictive accuracy. In contrast, BGWO_SA_Ens excels in identifying a moderate yet highly effective gene set, balancing feature reduction with high predictive performance. This underscores the advantage of BGWO_SA_Ens in handling high-dimensional, complex datasets typical in biological research. However, it’s worth noting that the increased complexity and computational demand of BGWO_SA_Ens could be seen as a drawback compared to more straightforward methods like LASSO. Overall, our findings suggest that while simpler methods have their merits, especially in less complex datasets, the sophisticated nature of BGWO_SA_Ens offers significant advantages in navigating the intricacies of genomic data for breast cancer research.

In the comparative analysis of different FS methods, the BGWO_SA_Ens algorithm demonstrated superior performance in terms of F1, PR-AUC, ROC-AUC, MCC, and BAc compared to BGWO_Ens, GA_Ens, LASSO, MCFS-IFS, and mRMR-IFS. BGWO_Ens and GA_Ens, despite selecting larger sets of features, did not match the performance of BGWO_SA_Ens. Additionally, the inclusion of MCFS-IFS and mRMR-IFS in the evaluation also provided a broader comparison basis. Notably, BGWO_SA_Ens excelled with an F1 score of 0.984, PR-AUC of 0.986, ROC-AUC of 0.977, MCC of 0.967, and BAc of 0.977 for the merged dataset, and similar high scores for GSE45827, as shown in Table 3. In contrast, LASSO’s performance was the lowest, and while MCFS-IFS and mRMR-IFS had varying results, they did not outperform BGWO_SA_Ens. This comparison underscores BGWO_SA_Ens’s ability to select a gene set that is not only moderate in size but also highly effective, as shown by the robust performance metrics across both datasets.

In our study, through meticulous methodology, the BGWO_SA_Ens algorithm selected a set of 1404 genes from the merged dataset and 1710 from the GSE45827 dataset. In parallel, we identified 164 differentially expressed genes (DEGs) from the merged dataset and 350 from the GSE45827 dataset. Crucially, the intersection of these four feature sets yielded 35 genes that were common to both datasets, which we designated as superior genes. This subset represents a highly significant group, as these genes are validated both through differential expression analysis and the advanced FS capabilities of the BGWO_SA_Ens algorithm. This intersection reinforces the validity of the selected genes and underscores the robustness of our approach in identifying key biomarkers for BC.

Additionally, our exploration of GO and KEGG enrichment analyses has revealed significant insights into the biological relevance of genes identified by our FS method. In the realm of biological processes, the enrichment results highlighted several key areas, with the “positive regulation of lipid localization” pathway being particularly notable. This pathway includes CD36, ADIPOQ, ACACB, CIDEA, LPL, and SPP1 genes, underscoring their potential role in BC biology. Similarly, in cellular components, the “collagen-containing extracellular matrix” component was identified as significant. This component features COL10A1, ITIH5, ADIPOQ, ADAMTS5, GPC3, SFRP1, and RARRES2 genes, pointing to their involvement in cancer-related cellular structures. In Molecular Functions, “sulfur compound binding” was determined as significant which implicating PCOLCE2, ADAMTS5, ACACB, SFRP1, and LPL genes.

Our KEGG pathway analysis further illuminated the importance of the “AMPK signaling pathway” in BC, with a notable association of genes such as CD36, ADIPOQ, ACACB, CIDEA, and LEP. This pathway is critical in regulating metabolic processes , including lipid metabolism and energy homeostasis, and has been linked to cancer development and progression [40]. The association of these genes with the AMPK signaling pathway emphasizes their potential role in modulating tumor growth, metabolism, and response to therapy in breast cancer biology.

In our comprehensive analysis, we identified several key genes that are potentially pivotal in the pathogenesis and progression of BC. Among them, CIDEA (Cell Death-Inducing DFFA-Like Effector A) is downregulated in breast tumors, suggesting its significant role in lipid metabolism and energy balance with implications for cancer cell survival and proliferation [57]. LEP (Leptin), identified as a superior downregulated gene in differential expression analysis, is recognized for its influence on energy regulation and a notable role in tumorigenesis, especially in BC. It contributes to cell proliferation and angiogenesis, key factors in cancer progression [58].

Another significant gene, acetyl-CoA carboxylase beta (ACACB), which is crucial for fatty acid oxidation and is downregulated in BC, is associated with increased survival and reduced drug resistance, highlighting its potential as a target for inhibiting tumor cell proliferation and metabolic reprogramming [59]. The Lipoprotein Lipase (LPL) gene stands out for its role in lipid metabolism and its potential involvement in providing fatty acids to cancer cells, thereby supporting their growth and survival [60]. ADIPOQ (Adiponectin), primarily known for its role in glucose regulation and fatty acid breakdown, has been observed to have anti-proliferative effects on BC cells, suggesting a protective role [61].

Our research has unveiled a range of additional genes with promising roles in BC, as determined through GO and KEGG enrichment analyses. These include RARRES2 (Retinoic Acid Receptor Responder 2) [62], AKR1C3 (Aldo-Keto Reductase Family 1 Member C3) [57], SPP1 (Secreted Phosphoprotein 1) [63], CIDEC (Cell Death-Inducing DFFA-Like Effector C) [64], CD36 (Cluster of Differentiation 36) [59], and MMP1 (Matrix Metallopeptidase 1) [65]. These genes contribute to the intricate molecular framework of BC, offering new avenues for exploration and potential therapeutic intervention.

Our findings underscore the crucial role of lipid metabolism disruptions in BC, particularly through the AMPK signaling pathway, indicating their importance in BC’s development and progression. The genes involved in these processes offer insights into BC’s mechanisms and pave the way for new therapeutic and biomarker development strategies. The comprehensive integration of GO and KEGG enrichment analyses with gene functions highlights BC’s complexity, advocating for a holistic understanding and targeted approach to this disease.

The detailed PPI network analysis has revealed key genes and functional modules, enhancing our understanding of BC’s intricate molecular interactions. This analysis, illustrated in figures like 12 and subnetworks in 13, identifies genes involved in lipid metabolism, cell cycle regulation, and EMT. These findings not only uncover underlying protein interactions but also suggest potential biomarkers and therapeutic targets, forming a foundation for future research in unraveling BC’s molecular complexities and advancing precision medicine.

Our study’s focus on drug-gene interactions has shed light on potential BC treatments, particularly highlighting topoisomerase II alpha (TOP2A) [66] inhibitors and their role in BC’s development and progression. Drugs such as vincristine, teniposide, mitoxantrone, etoposide, epirubicin, idarubicin, daunorubicin, amsacrine, paclitaxel, dexrazoxane, doxorubicin, and fluorouracil all target TOP2A, indicating its crucial role in either the development or progression of BC. This examination reveals complex interplays between genes and drugs, suggesting pathways for therapeutic exploitation and strategies to hinder cancer progression. It points towards a personalized treatment approach in BC, targeting specific genes with multiple drugs to tailor treatments. Overall, this study demonstrates the effectiveness of computational techniques in identifying significant BC biomarkers, emphasizing the need for further experimental validation and the potential to extend these methods to other cancers for precision medicine advancements.

## Conclusion

In conclusion, this study demonstrates the efficacy of an innovative ML workflow in identifying robust biomarkers and therapeutic targets for BC. By synergizing metaheuristic optimization, ensemble learning, differential expression analysis, and network biology, a panel of 35 superior genes was revealed. These genes were not only differentially expressed in BC but also integral to predictive modeling, affirming their significance.

Collectively, this multipronged informatics approach overcomes key challenges in biomarker discovery. It enhances model accuracy, generalizability, and biological relevance compared to prevailing techniques. The findings propel novel possibilities for diagnostic, prognostic and therapeutic innovation in BC. Moving forward, experimental validation of the identified genes and drug targets will further cement this study’s contributions to precision oncology.

The framework developed is widely applicable for mining biomarkers from multifaceted biomedical data. Furthermore, the proposed BGWO_SA_Ens algorithm sets a new standard for efficient FS in ML pipelines. By synergizing the strengths of optimization, ensembles, and differential expression, more refined and biologically insightful gene signatures can be derived. This advances personalized medicine across diverse diseases. In summary, this research underscores the translational potential of informatics in augmenting biomarker discovery and augmenting our comprehension of complex diseases.

## Data Availability

The datasets examined in this study can be found in the GEO repository, under the NCBI GEO accession numbers GSE42568 [67] and GSE10810 [68]. All codes associated with this paper are available at https://github.com/mornejd/Refining-Breast-Cancer-Biomarker-Discovery-BGWO_SA_Ens. Also, for any additional inquiries contact the corresponding author, Mohammad Fathian (fathian@iust.ac.ir).
